# Hyperchloremia Is Associated With Poorer Outcome in Critically Ill Stroke Patients

**DOI:** 10.3389/fneur.2018.00485

**Published:** 2018-07-03

**Authors:** Kaibin Huang, Yanhong Hu, Yongming Wu, Zhong Ji, Shengnan Wang, Zhenzhou Lin, Suyue Pan

**Affiliations:** Department of Neurology, Nanfang Hospital, Southern Medical University, Guangzhou, China

**Keywords:** hyperchloremia, neurocritical care, mortality, poor prognosis, fluid management

## Abstract

**Background and Purpose:** This study aims to explore the cause and predictive value of hyperchloremia in critically ill stroke patients.

**Materials and Methods:** We conducted a retrospective study of a prospectively collected database of adult patients with first-ever acute ischemic stroke (AIS) or intracerebral hemorrhage (ICH) admitted to the neurointensive care unit (NICU) of a university-affiliated hospital, between January 2013 and December 2016. Patients were excluded if admitted beyond 72 h from onset, if they required neurocritical care for less than 72 h, and were treated with hypertonic saline within 72 h or had creatinine clearance less than 15 mL/min.

**Results:** Of 405 eligible patients, the prevalence of hyperchloremia ([Cl^−^] ≥ 110 mmol/L) was 8.6% at NICU admission ([Cl^−^]_0_) and 17.0% within 72 h ([Cl^−^]_max_). Thirty-eight (9.4%) patients had new-onset hyperchloremia and 110 (27.1%) had moderate increase in chloride (Δ[Cl^−^] ≥ 5 mmol/L; Δ[Cl^−^] = [Cl^−^]_max_ − [Cl^−^]_0_) in the first 72 h after admission, which were found to be determined by the sequential organ failure assessment score in multivariate logistic regression analysis. Neither total fluid input nor cumulative fluid balance had significant association with such chloride disturbance. New-onset hyperchloremia and every 5 mmol/L increment in Δ[Cl^−^] were both associated with increased odds of 30-day mortality and 6-month poor outcome, although no independent significance was found in multivariate models.

**Conclusion:** Hyperchloremia tends to occur in patients more severely affected by AIS and ICH. Although no independent association was found, new-onset hyperchloremia and every 5 mmol/L increment in Δ[Cl^−^] were related to poorer outcome in critically ill AIS and ICH patients.

**Subject terms:** clinical studies, intracranial hemorrhage, ischemic stroke, mortality/survival, quality and outcomes.

## Introduction

Critically ill acute ischemic stroke (AIS) and intracerebral hemorrhage (ICH) patients account for most admissions to the neurointensive care unit (NICU) (1). These diseases heavily burden the family and society, making treatment and outcome prediction particularly important. Fluid management aiming at maintaining adequate cerebral blood flow and oxygenation plays a crucial role in the management of these patients (2). Because of the propensity to cause brain edema by hypo-osmolar balanced crystalloids, hypertonic saline or normal saline (0.9% saline solution) are more frequently used in brain-injured patients (3). However, concerns have been raised that both hypertonic saline (4, 5) and large-volume infusion of normal saline (6, 7) may raise the risk of hyperchloremia.

Hyperchloremia has been reported to be associated with increased hospital mortality and negative outcome in critically ill patients (8), with severe sepsis and septic shock (9, 10), as well as after surgery and trauma (11, 12). In patients with ICH, a recent study demonstrated higher rates of in-hospital mortality in those who developed moderate hyperchloremia during treatment with continuous intravenous infusion of 3% hypertonic saline, with moderate hyperchloremia independently predicting in-hospital mortality (13). Another study in patients with subarachnoid hemorrhage found a strong association between hyperchloremia and acute kidney injury (AKI) as well as AKI and mortality (14). However, whether routinely used normal saline raises the risk of hyperchloremia in critically ill stroke patients remains largely unknown. Besides, the cause and predictive value of hyperchloremia in these patients requires further evaluation.

In this study, we aimed to identify risk factors that related to the development of hyperchloremia in critically ill patients with AIS and ICH, with particular focus to the amount of normal saline infusion. The influence of hyperchloremia and chloride fluctuation on patient outcome was evaluated as well.

## Materials and methods

### Study design and participants

**Figure 1:**
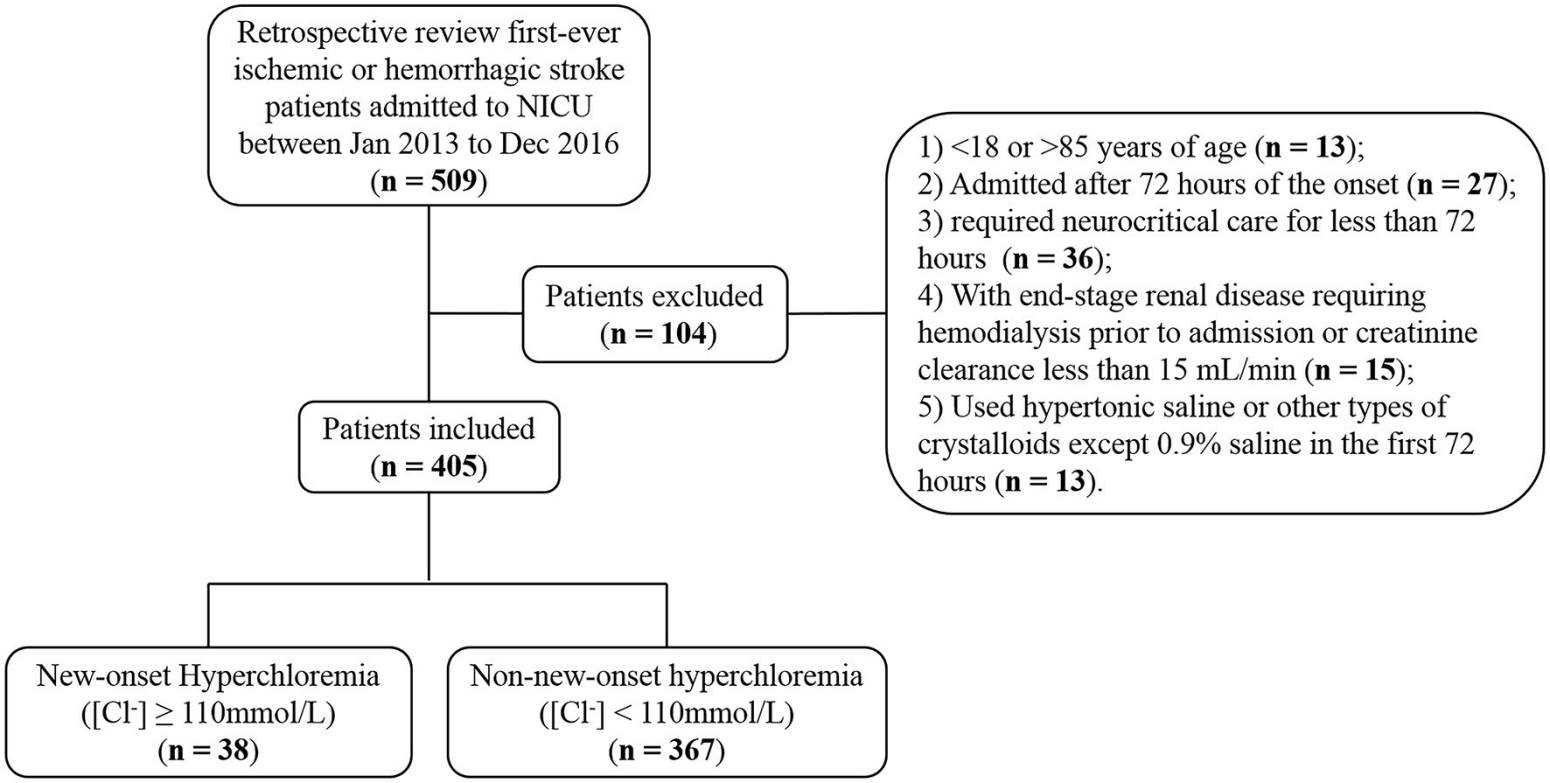
Patient inclusion flowchart.

The data that support the findings of this study are available from the corresponding author upon reasonable request. We conducted a retrospective study of a prospectively collected database of adult patients with first-ever AIS or ICH admitted to the NICU of Nanfang Hospital, a university-affiliated academic hospital, between January 2013 and December 2016 (Figure 1). Patients were excluded if they were younger than 18 or older than 85, admitted beyond 72 h of the onset, required neurocritical care for less than 72 h, had premorbid disability [modified Rankin Scale (mRS) > 1] or end-stage renal disease requiring hemodialysis or creatinine clearance less than 15 mL/min. To explore the association between normal saline infusion and hyperchloremia, we also excluded patients who received hypertonic saline (3% or 10%) or other types of crystalloids except 0.9% saline during the first 72 h of NICU admission. The study proposal was approved by the hospital's ethics committee for clinical research. Informed consent was waived by the review board, because this study was observational, retrospective, and all data were fully de-identified.

### Study variables

Electronic medical records were carefully reviewed to collect patient demographics, previous medical history, laboratory values, and ICD-10-based final diagnoses. Since the concentration of serum chloride ([Cl^−^]) shifts constantly, we defined the [Cl^−^] at the time of NICU admission as [Cl^−^]_0_ (baseline chloride) and the following maximum [Cl^−^] in the first 72 h as [Cl^−^]_max_. Hyperchloremia was defined as [Cl^−^] ≥ 110 mmol/L (9). The increase in serum chloride (Δ[Cl^−^] = [Cl^−^]_max_ − [Cl^−^]_0_) was calculated, and Δ[Cl^−^] ≥ 5 mmol/L was regarded as a moderate increase in chloride (8).

Glasgow Coma Scale (GCS) scores were extracted from the first neurological examination at NICU admission. The total score of Sequential Organ Failure Assessment (SOFA) (15) was obtained according to its corresponding parameters in the first 24 h of NICU admission. The requirement of vasopressor agents and mechanical ventilation within 24 h of NICU admission were also recorded. Total fluid input within the first 72 h was measured, and cumulative fluid balance (CFB) was calculated based on total fluid input minus output during this period. In order to explore the correlation between hyperchloremia and AKI, we used the kidney disease improving global outcomes (KDIGO) as diagnostic criteria of AKI (16).

### Study outcomes

Primary end points were 30-day mortality and 6-month poor outcome, with the latter defined as mRS of 4–6. Information on survival and functional outcome were obtained through telephone review by a trained neurologist blinded to the study data.

### Statistical analysis

Categorical variables were presented as number (%) and were compared using the two-sided chi-square test or Fisher's exact test. Continuous data were presented as mean ± standard deviation (SD) or median [25–75% interquartile range (IQR)] and compared by Student's *t*-test or Mann–Whitney *U* test, as appropriate.

To explore risk factors associated with the development of hyperchloremia, patients with normal [Cl^−^] at the time of NICU admission but who developed hyperchloremia in the first 72 h of NICU stay were defined as new-onset hyperchloremia. The whole study sample was then divided into the new-onset hyperchloremia and non-new-onset hyperchloremia subgroups. Univariate analysis was first performed, and candidate variables that had a *p*-value less than 0.05 were drawn into multivariate logistic regression model. Candidate variables included demographic data (age, gender), comorbidity (baseline creatinine, diabetes, hypertension, heart failure), indicators of critical illness (AKI, NIHSS, GCS, SOFA, base deficit, vasopressors, mechanical ventilation), and fluid management (total fluid input and CFB in the first 72 h) (9). Only one of the two variables was included in the event of collinearity between variables. The 95% confident intervals (CIs) reported for the logistic regression odds ratios (ORs) were calculated by the Wald estimation. The risk factors of moderate increase in chloride (Δ[Cl^−^] ≥ 5 mmol/L) were evaluated using similar statistical methods as well.

To evaluate the influence of hyperchloremia on patient outcome, the associations between 30-day mortality or 6-month poor outcome (dependent variables) and (1) [Cl^−^] at the time of NICU admission ([Cl^−^]_0_), (2) maximum [Cl^−^] at the first 72 h of NICU stay [Cl^−^]_max_), (3) Δ[Cl^−^] (Δ[Cl^−^] = [Cl^−^]_max_ − [Cl^−^]_0_), (4) new-onset hyperchloremia were examined using logistic regression analysis. The associations between patient outcomes and the independent variables of interest ([Cl^−^]_0_, [Cl^−^]_max_, Δ[Cl^−^] and new-onset hyperchloremia) were further examined, separately, in multivariate logistic regression models that adjusted for confounders known to be associated with hospital mortality, as described in the above section. All analyses were performed using SPSS, version 23.0 (SPSS, Chicago, IL). A two-sided *p*-value less than 0.05 was considered to be statistically significant.

## Results

Of 509 patients screened for eligibility, 405 satisfied inclusion and exclusion criteria (Figure 1). Of those, 250 (61.7%) patients were diagnosed with AIS and 155 (38.3%) were ICH. There were 35 (8.6%) patients with hyperchloremia at the time of NICU admission ([Cl^−^]_0_ ≥ 110 mmol/L), and the number increased to 69 (17.0%) within 72 h after admission ([Cl^−^]_max_ ≥ 110 mmol/L). Of note, 38 (9.4%) patients had new-onset hyperchloremia in the first 72 h after NICU admission (Figure 1). The median [Cl^−^] was 112 mmol/L (111^−^115 mmol/L) in the new-onset hyperchloremic subgroup and 104 mmol/L (102–107 mmol/L) in the non-new-onset hyperchloremic subgroup. Baseline demographics and clinical characteristics of these two subgroups were summarized in Table 1. The patients with new-onset hyperchloremia had lower GCS, higher NIHSS, and SOFA scores. Moreover, these patients required more vasopressors and mechanical ventilation when compared with those without new-onset hyperchloremia within 72 h of NICU admission. However, neither total fluid input nor CFB had significant association with new-onset hyperchloremia (Table 1).

**Table 1:** Baseline demographics and clinical characteristics stratified by new-onset hyperchloremia ([Cl^−^] ≥ 110 mmol/L) or not ([Cl^−^] < 110 mmol/L) within 72 h of NICU admission.

**Variable**	**New-onset HC (*n* = 38)**	**Non-new-onset HC (*n* = 367)**	***p*-Value**
Demographics			
Age, year, median (IQR)	56 (46–69)	62 (50–72)	0.188
Male, *n* (%)	27 (71.1%)	251 (68.4%)	0.447
Chronic conditions			
Baseline serum creatinine, μmol/L, median (IQR)	90 (73–115)	82 (65–104)	0.135
Hypertension, *n* (%)	24 (63.2%)	236 (64.3%)	0.509
Diabetes mellitus, *n* (%)	8 (21.1%)	71 (19.3%)	0.471
Heart disease, *n* (%)	8 (21.1%)	63 (17.2%)	0.341
Critical indicators on NICU admission			
BE, mmol/L, median (IQR)	−1.3 (−4.0 to 1.65)	−0.2 (−2.0 to 1.7)	0.083
GCS, median (IQR)	9 (6–11)	11 (9–12)	0.001
NIHSS, median (IQR)	17 (11–22)	12 (7–17)	0.002
SOFA, median (IQR)	8 (4–10)	4 (2–6)	<0.001
Laboratory indicators			
Lactate, mmol/L, median (IQR)	2.8 (2.1–3.2)	2.3 (2.0–3.1)	0.392
Albumin, g/L, median (IQR)	38 (33–41)	39 (34–43)	0.240
Fluid indicators within 72 h			
Total fluid input (with enteral nutrition) within 72 h, L, median (IQR)	7.7 (6.0–9.6)	7.1 (6.1–8.2)	0.076
Total fluid input (without enteral nutrition) within 72 h, L, median (IQR)	4.2 (2.8–6.1)	3.6 (2.7–4.8)	0.074
Cumulative fluid balance within 72 h, L, mean ± SD	1.8 ± 1.6	1.7 ± 1.5	0.969
Vasopressor, *n* (%)	9 (23.7%)	22 (6.0%)	0.001
Mechanical ventilation, *n* (%)	17 (44.7%)	75 (20.4%)	0.001
Acute kidney injury, *n* (%)	7 (18.4%)	31 (8.4%)	0.052

*HC, hyperchloremia ([Cl^−^] ≥ 110 mmol/L); SD, standard deviation; BE, base excess; NIHSS, National Institutes of Health Stroke Scale; GCS, Glasgow Coma Scale; SOFA, Sequential Organ Failure Assessment; IQR, interquartile range*.

Since GCS is included in SOFA and had collinearity with NIHSS, it was omitted from multivariate logistic analysis. In a multivariate model evaluating risk factors that related to the development of new-onset hyperchloremia, SOFA was identified as the only independent variable that associated with new-onset hyperchloremia (Table 2). Furthermore, SOFA and mechanical ventilation were found to be the risk factors of moderate increase in chloride (Δ[Cl^−^] ≥ 5 mmol/L) in the first 72 h in both univariate and multivariate logistic analysis (Supplementary Tables S1, S2).

**Table 2:** Univariate and multivariate logistic regression analysis for risk factors of new-onset hyperchloremia ([Cl^−^] ≥ 110 mmol/L).

**Variable**	**Univariate analysis**			Multivariate analysis^*^		
	**OR**	**95% CI**	***p*-Value**	**OR**	**95% CI**	***p*-Value**
NIHSS	1.085	1.031–1.143	0.002	–	–	–
SOFA	1.259	1.146–1.384	<0.001	1.259	1.146–1.384	<0.001
Vasopressor	4.867	2.053–11.537	<0.001	–	–	–
Mechanical ventilation	3.152	1.584–6.271	0.001	–	–	–

NIHSS, National Institutes of Health Stroke Scale; SOFA, Sequential Organ Failure Assessment; OR, odds ratio; CI, confidence interval.

* *In multivariate logistic regression, only parameter with statistical significance was shown*.

During follow up, 61 (15.1%) patients died within 30 days after admission and 236 (58.3%) patients achieved good outcome at 6 months. Results showed that patients with new-onset hyperchloremia were associated with a 158% increase in odds for 30-day mortality (OR = 2.58; 95% CI, 1.21–5.53; *p* = 0.015) (Table 3). In addition, each 5 mmol/L increase in [Cl^−^]_0_, [Cl^−^]_max_, and Δ[Cl^−^] was associated with a 50, 66, and 55% increase in odds for 30-day mortality, respectively (Table 3). However, none of these changes of chloride showed independent association with 30-day mortality in multivariate logistic analysis, while age, base excess, SOFA, and mechanical ventilation were found to be significant (Table 3). In terms of 6-month functional outcome, new-onset hyperchloremia, [Cl^−^]_max_ and Δ[Cl^−^] were all associated with increased risk of poor outcome, with an OR (95% CI) of 3.394 (1.660–6.941), 1.425 (1.205–1.685), and 1.383 (1.143–1.674), respectively (Table 4). Nevertheless, these three indicators were not independently related to 6-month outcome in multivariate models, where age, NIHSS, and SOFA were independent risk factors of poor outcome (Table 4). The detailed statistical results were summarized in Supplementary Tables S3, S4.

**Table 3:** Univariate and multivariate logistic regression analysis of risk factors associated with 30-day mortality.

**Variable**	**Univariate analysis**		**Multivariate analysis^†^**	
	**Odds ratio (95% CI)**	***p*-Value**	**Odds ratio (95% CI)**	***p*-Value**
Age	1.026 (1.005–1.048)	0.016	1.035 (1.006–1.064)	0.016
Baseline serum creatinine	1.007 (1.003–1.012)	0.001	–	–
Base excess	0.788 (0.720–0.863)	<0.001	0.867 (0.782–0.960)	0.006
NIHSS	1.124 (1.075–1.176)	<0.001	–	–
SOFA	1.733 (1.526–1.969)	<0.001	1.540 (1.324–1.792)	<0.001
Albumin	0.925 (0.885–0.966)	<0.001	–	–
Vasopressors	17.535 (7.713–39.867)	<0.001	–	–
Mechanical ventilation	15.961 (8.438–30.191)	<0.001	2.705 (1.183–6.189)	0.018
Acute kidney injury	10.099 (4.921–20.722)	<0.001	–	–
New-onset hyperchloremia^*^	2.583 (1.206–5.533)	0.015	–	–
[Cl^−^]_0_ (per 5 mmol/L)^*^	1.502 (1.168–1.932)	0.002	–	–
[Cl^−^]_max_ (per 5 mmol/L)^*^	1.657 (1.368–2.007)	<0.001	–	–
Δ[Cl^−^] (per 5 mmol/L)^*^	1.552 (1.235–1.951)	<0.001	–	–

* The indicators of chloride were drawn into multivariable logistic analysis separately.

† *Since age, base excess, SOFA, and mechanical ventilation were consistently found to be independent factors associated with 30-day mortality when each indicator of chloride was included, their odds ratio value and p-value were given when new-onset hyperchloremia was drawn in multivariate analysis only. GCS was not included in the multivariate model because of collinearity with the NIHSS. Serum creatinine was not included in the multivariate model because of collinearity with the acute kidney injury. CI, confidence interval*.

**Table 4:** Univariate and multivariate logistic regression analysis of risk factors associated with 6-month poor outcome (mRS ≥ 4).

**Variable**	**Univariate analysis**		**Multivariate analysis^†^**	
	**Odds ratio (95% CI)**	***p*-Value**	**Odds ratio (95% CI)**	***p*-Value**
Age	1.032 (1.016–1.047)	<0.001	1.038 (1.019–1.057)	<0.001
Diabetes mellitus	1.669 (1.018–2.736)	0.042	–	–
NIHSS	1.143 (1.103–1.184)	<0.001	1.087 (1.041–1.136)	<0.001
SOFA	1.439 (1.323–1.565)	<0.001	1.308 (1.191–1.437)	<0.001
Albumin	0.926 (0.894–0.959)	<0.001	–	–
Vasopressors	11.028 (3.780–32.172)	<0.001	–	–
Mechanical ventilation	5.544 (3.307–9.293)	<0.001	–	–
Acute kidney injury	3.394 (1.660–6.941)	0.001	–	–
New-onset hyperchloremia^*^	3.394 (1.660–6.941)	0.001	–	–
[Cl^−^]_0_ (per 5 mmol/L)^*^	1.203 (0.995–1.453)	0.056	–	–
[Cl^−^]_max_ (per 5 mmol/L)^*^	1.425 (1.205–1.685)	<0.001	–	–
Δ[Cl^−^] (per 5 mmol/L)^*^	1.383 (1.143–1.674)	0.001	–	–

* The indicators of chloride were drawn into multivariable logistic analysis separately.

† *Since age, NIHSS, and SOFA were consistently found to be independent factors associated with 30-day mortality when each indicator of chloride was included, their odds ratio value, and p-value were given when new-onset hyperchloremia was drawn in multivariate analysis only. GCS was not included in the multivariate model because of collinearity with the NIHSS. CI, confidence interval*.

## Discussion

In this study, we showed that the prevalence of hyperchloremia in critically ill stroke patients increased from 8.6% at NICU admission to 17.0% in the first 72 h. The SOFA score was identified as an independent risk factor of both new-onset hyperchloremia and moderate increase in chloride (Δ[Cl^−^] ≥ 5 mmol/L) after NICU admission. For the infusion of normal saline, neither total fluid input nor CFB was significantly related to new-onset hyperchloremia or moderate increase in chloride. Although no independent significance was found in multivariate models, both new-onset hyperchloremia and every 5 mmol/L increment in Δ[Cl^−^] were associated with increased odds of 30-day mortality or 6-month poor outcome.

Chloride is the most abundant anion in the extracellular fluid, constituting approximately one-third of plasma tonicity, and plays an essential role in various body functions including the regulation of body fluids, electrolyte balance, acid-base status, muscular activity, osmosis, and immunomodulation (17). While the concentration of chloride shifts constantly during hospitalization, chloride receives much less attention than other routinely measured electrolytes until recent reports investigated the influence of hyperchloremia on hospital mortality and AKI (18).

In a retrospective study, hyperchloremia and moderate increase in serum chloride (Δ[Cl^−^] ≥ 5 mmol/L) were both found to be independently associated with AKI in severe sepsis and septic shock patients (10). (9) revealed that hyperchloremia at 72 h of ICU stay (Cl_72_) were related to all-cause hospital mortality and every 5 mmol/L increment in Cl_72_ or Δ[Cl^−^] was, respectively, associated with a 27 or 37% increase in the odds for hospital mortality in those critically ill septic patients who were already hyperchloremic on admission to the ICU. (19) conducted a respective study on 76,719 hospitalized patients and found that both serum chloride alterations on admission and post-admission serum chloride increase predicted poor outcome. Similar to these studies, we observed that both new-onset hyperchloremia or every 5 mmol/L increment in Δ[Cl^−^] were associated with increased odds of poor outcome in critically ill stroke patients, although these two variables did not show independent significance in multivariate models. The possible explanation could be that the prevalence of hyperchloremia in our subjects (17.0% in the first 72 h) was much lower than that in other patient groups [65.3% (13) in patients with ICH and treated with hypertonic saline; 40.8% ((10)) and 31.7% (9) in patients with septic shock].

Hyperchloremia may result from disease processes or clinical interventions (18). In the present study, we observed that the main determinant of hyperchloremia in critically ill stroke patients was the disease severity, as reflected by the SOFA score. While previous studies have shown a strong association between hyperchloremia and administration of chloride-rich fluids (18), such as 0.9% saline (20), we did not find a significant association between normal saline infusion and new-onset hyperchloremia or moderate increase in chloride. The plausible explanation was that we only observed the chloride fluctuation within the first 72 h after NICU admission. Extending the observation period might result in different findings. Besides, we have excluded patients who had end-stage renal disease requiring hemodialysis or creatinine clearance less than 15 mL/min at the screening, which may decrease the risk of chloride retention. Finally, compared with the treatment in septic shock, the infused volume of normal saline was much lower in our study subjects, which might have weakened the association between normal saline infusion and chloride fluctuation. Although other balanced crystalloid fluid such Plasma-Lyte (Na, 140 mmol/L; Cl, 98 mmol/L) has been reported to cause less hyperchloremia (21), normal saline remains to be the first choice in most clinical scenarios (22). The encouraging findings from two recent trials that lactated Ringer's solution or Plasma-Lyte A reduced hospital death or adverse kidney events in contrast to saline in critically ill (23) and non-critically ill (24) adult patients may have the potential to change current practice.

Our results should be interpreted with caution. First, the retrospective nature of this study make it susceptible to selection and information bias. Second, we were unable to assess all potentially relevant variables. Still, the candidate variables selected in the present study were mostly in line with two representative studies (9, 14). Third, to explore the correlation between normal saline infusion and chloride disturbance, we have excluded patients who were treated with hypertonic saline within 72 h, which might have underestimated the prevalence of hyperchloremia in our study subjects. Fourth, the amount of chloride add through other chlorinated fluid such as potassium chloride and enteral nutrients were not evaluated. Nevertheless, the contribution of potassium chloride to serum chloride is far less than normal saline, and the supplement of enteral nutrients to patients were strictly conducted according to the operating guidelines (25, 26).

## Conclusion

Hyperchloremia is not rare and tends to occur in patients more severely affected by AIS and ICH. While no independent association was found, new-onset hyperchloremia and every 5 mmol/L increment in Δ[Cl^−^] within 72 h of NICU admission were associated with an increased odds of all cause 30-day mortality and 6-month poor prognosis in critically ill stroke patients. This study indicates that hyperchloremia has clinical importance as an indicator of prognosis in critically ill patients. Prospective study with rigorous design should be help to further explore the casual relationship between hyperchloremia and clinical outcome.

## Ethics statement

This study was approved by the Nanfang Hospital's ethics committee on clinical research. Informed consent was waived by the review board because of the pure observational and retrospective nature of the study.

## Author contributions

SP contributed to study conception and design. KH participated in study conception and design, and helped to draft and revise the manuscript. YH collected data and drafted the manuscript. YW and ZJ performed statistical analysis. SW and ZL participated in patient follow up and helped to revise the manuscript. All authors made substantial contribution, and read and approved the final version of the manuscript.

### Conflict of interest statement

The authors declare that the research was conducted in the absence of any commercial or financial relationships that could be construed as a potential conflict of interest.
