# Supplementary material for: Hyperchloremia Is Associated With Poorer Outcome in Critically Ill Stroke Patients
Source: Front Neurol. 2018 Jul 3;9:485. doi: 10.3389/fneur.2018.00485 (PMC6037722; doi:10.3389/fneur.2018.00485)
Supplement: Supplementary file 1 [file Table_1.DOCX]

**Supplementary Table 1** Baseline Demographics and Clinical Characteristics Stratified by Moderate Increase in Chloride (△[Cl^-^] ≥ 5 mmol/L) or Not (△[Cl^-^] < 5 mmol/L) in the First 72 Hours of NCU Admission.

| Variable | △[Cl^-^] ≥ 5 mmol/L **(n = 110)** | **△[Cl-] < 5 mmol/L**  **(n = 295)** | *P* value |
| --- | --- | --- | --- |
| Demographics |  |  |  |
| Age, yr, median (IQR) | 60 (48-70) | 62 (50-73) | 0.157 |
| Male, n (%) | 75 (68.2%) | 203 (68.8%) | 0.497 |
| Chronic conditions |  |  |  |
| Baseline serum creatinine, μmol/L, median (IQR) | 90 (67-116) | 62 (50-73) | 0.010 |
| Hypertension, n (%) | 75 (68.2%) | 185 (62.7%) | 0.183 |
| Diabetes mellitus, n (%) | 26 (23.6%) | 53 (18.0%) | 0.128 |
| Heart disease, n (%) | 20 (18.2%) | 51 (17.3%) | 0.469 |
| Critical indicators on NCU admission |  |  |  |
| BE, mmol/L, median (IQR) | -0.3 (-2.7-1.7) | -0.4 (-0.2-1.8) | 0.381 |
| GCS, median (IQR) | 10 (6-11) | 11 (9-12) | 0.004 |
| NIHSS, median (IQR) | 17 (12-22) | 13 (9-16) | <0.001 |
| SOFA, median (IQR) | 6 (3-9) | 3 (2-6) | <0.001 |
| Laboratory indicators |  |  |  |
| Lactate, mmol/L, median (IQR) | 2.6 (2.1-3.1) | 2.3 (2.0-3.2) | 0.307 |
| Albumin, g/L, mean ± SD | 38.2 ± 7.0 | 38.4 ± 5.9 | 0.810 |
| Fluid indicators within 72 hours |  |  |  |
| Total fluid input (with enteral nutrition) within 72 hours, L, median (IQR) | 7.7 (6.1-9.0) | 7.1 (6.1-8.1) | 0.056 |
| Total fluid input (without enteral nutrition) within 72 hours, L, median (IQR) | 4.3 (2.6-5.5) | 3.7 (2.7-4.8) | 0.070 |
| Cumulative fluid balance within 72 hours, L, mean ± SD | 1.8 ± 1.8 | 1.7 ± 1.4 | 0.468 |
| Vasopressors, n (%) | 19 (17.3%) | 12 (4.1%) | < 0.001 |
| Mechanical ventilation, n (%) | 49 (44.5%) | 43 (14.6%) | < 0.001 |
| Acute Kidney Injury, n (%) | 22 (20.0%) | 16 (5.4%) | < 0.001 |

HC, hyperchloremia ([Cl^-^] ≥ 110 mmol/L); SD, standard deviation; BE, base excess; GCS, Glasgow coma scale; NIHSS, National Institute of Health stroke scale; SOFA, sequential organ failure assessment; IQR, interquartile range.
