# Supplementary material for: Hyperchloremia Is Associated With Poorer Outcome in Critically Ill Stroke Patients
Source: Front Neurol. 2018 Jul 3;9:485. doi: 10.3389/fneur.2018.00485 (PMC6037722; doi:10.3389/fneur.2018.00485)
Supplement: Supplementary file 2 [file Table_2.DOCX]

**Supplementary Table 2** Univariate and Multivariate Logistic Regression Analysis for Risk Factors of Moderate Increase in Chloride (△[Cl^-^] ≥ 5 mmol/L).

| **Variable** | **Univariate analysis** | | |  | **Multivariate logistic regression** | | |
| --- | --- | --- | --- | --- | --- | --- | --- |
|  | **OR** | **95% CI** | ***P* value** |  | **OR** | **95% CI** | ***P* value** |
| Baseline serum creatinine | 1.003 | 1.000-1.006 | 0.073 |  | - | - | - |
| NIHSS | 1.036 | 1.002-1.070 | 0.037 |  | - | - | - |
| SOFA | 1.342 | 1.230-1.464 | < 0.001 |  | 1.236 | 1.111-1.375 | < 0.001 |
| Vasopressor or inotrope | 0.203 | 0.095-0.434 | < 0.001 |  | - | - | - |
| Mechanical ventilation | 0.212 | 0.129-0.349 | < 0.001 |  | 2.204 | 1.153-4.213 | 0.017 |
| Acute Kidney Injury | 4.359 | 2.193-8.666 | < 0.001 |  | - | - | - |

NIHSS, National Institute of Health stroke scale; SOFA, sequential organ failure assessment; OR, odds ratio; CI, confidence interval. In multivariate logistic regression, only parameters with statistical significance were shown.
