# Supplementary material for: Hyperchloremia Is Associated With Poorer Outcome in Critically Ill Stroke Patients
Source: Front Neurol. 2018 Jul 3;9:485. doi: 10.3389/fneur.2018.00485 (PMC6037722; doi:10.3389/fneur.2018.00485)
Supplement: Supplementary file 3 [file Table_3.DOCX]

**Supplementary Table 3** Baseline Demographics and Clinical Characteristics Stratified by Survivors and Non-Survivors in the First 30 Days of NCU Admission.

| Variable | Survivors  **(n = 344)** | **Non-survivors**  **(n = 61)** | *P* value |
| --- | --- | --- | --- |
| Demographics |  |  |  |
| Age, yr, median (IQR) | 61 (49-71) | 66 (57-74) | 0.011 |
| Male, n (%) | 236 (68.6) | 42 (68.9) | 0.549 |
| Chronic conditions |  |  |  |
| Baseline serum creatinine, μmol/L, median (IQR) | 81 (65-100) | 91 (71-125) | 0.006 |
| Hypertension, n (%) | 218 (63.4) | 42 (68.9) | 0.251 |
| Diabetes mellitus, n (%) | 62 (18.0) | 17 (27.0) | 0.057 |
| Heart disease, n (%) | 60 (17.4) | 11 (18.0) | 0.517 |
| Critical indicators on NCU admission |  |  |  |
| BE, mmol/L, mean ± SD | 0.1 ± 3.0 | -2.6 ± 4.0 | < 0.001 |
| NIHSS, median (IQR) | 12 (7-16) | 18 (12-22) | < 0.001 |
| GCS, median (IQR) | 11 (9-12) | 9 (6-11) | < 0.001 |
| SOFA, median (IQR) | 3 (2-6) | 9 (7-12) | < 0.001 |
| Laboratory indicators |  |  |  |
| Lactate, mmol/L, median (IQR) | 2.3 (2.0-3.0) | 3.0 (2.0-3.4) | 0.404 |
| Albumin, g/L, median (IQR) | 39 (35-43) | 35 (32-41) | < 0.001 |
| Fluid indicators within 72 hours |  |  |  |
| Total fluid input (with enteral nutrition) within 72 hours, L, median (IQR) | 7.1 (6.1-8.3) | 7.8 (6.3-9.0) | 0.149 |
| Total fluid input (without enteral nutrition) within 72 hours, L, median (IQR) | 4.3 (2.8-5.5) | 3.6 (2.6-4.8) | 0.136 |
| Cumulative fluid balance 72 hours, L, mean ± SD | 1.7 ± 1.4 | 1.7 ± 2.2 | 0.826 |
| Vasopressor or inotrope, n (%) | 10 (2.9) | 21 (34.4) | < 0.001 |
| Mechanical ventilation, n (%) | 48 (14.0) | 44 (72.1) | < 0.011 |
| Acute Kidney Injury, n (%) | 17 (4.9%) | 21 (34.4%) | < 0.001 |
| Chloride indicators |  |  |  |
| New-onset hyperchloremia, n (%) | 27 (7.8) | 11 (18) | 0.016 |
| [Cl^-^]_0_, mmol/L, median (IQR) | 103 (100-106) | 104 (100-110) | 0.058 |
| [Cl^-^]_max_, mmol/L, median (IQR) | 104 (102-108) | 110 (103-115) | < 0.001 |
| Δ[Cl^-^] ≥ 5 mmol/L, n (%) | 81 (23.5) | 29 (47.5) | < 0.001 |

HC, hyperchloremia ([Cl^-^] ≥ 110 mmol/L); SD, standard deviation; BE, base excess; GCS, Glasgow coma scale; APACHE II, acute physiology and chronic health evaluation II; SOFA, sequential organ failure assessment; IQR, interquartile range.
