# Supplementary material for: Hyperchloremia Is Associated With Poorer Outcome in Critically Ill Stroke Patients
Source: Front Neurol. 2018 Jul 3;9:485. doi: 10.3389/fneur.2018.00485 (PMC6037722; doi:10.3389/fneur.2018.00485)
Supplement: Supplementary file 4 [file Table_4.DOCX]

**Supplementary Table 4** Baseline Demographics and Clinical Characteristics Stratified by 6-Month Good (mRS ≤ 3) or Poor Outcome (mRS ≥ 4).

| Variable | Good outcome  **(n = 236)** | **Poor outcome**  **(n = 169)** | *P* value |
| --- | --- | --- | --- |
| Demographics |  |  |  |
| Age, yr, median (IQR) | 59 (48-69) | 65 (55-74) | < 0.001 |
| Male, n (%) | 71 (30.1) | 56 (33.1) | 0.293 |
| Chronic conditions |  |  |  |
| Baseline serum creatinine, μmol/L, median (IQR) | 83 (66-103) | 83 (65-107) | 0.648 |
| Hypertension, n (%) | 148 (62.7) | 112 (66.3) | 0.264 |
| Diabetes mellitus, n (%) | 38 (16.1) | 41 (24.3) | 0.028 |
| Heart disease, n (%) | 38 (16.1) | 33 (19.5) | 0.223 |
| Critical indicators on NCU admission |  |  |  |
| BE, mmol/L, mean ± SD | 0 (-1.9-1.8) | -0.6 (-2.4-1.7) | 0.091 |
| NIHSS, median (IQR) | 10 (6-15) | 16 (12-20) | < 0.001 |
| GCS, median (IQR) | 11 (10-14) | 10 (6-11) | < 0.001 |
| SOFA, median (IQR) | 3 (2-5) | 7 (3-9) | < 0.001 |
| Laboratory indicators |  |  |  |
| Lactate, mmol/L, median (IQR) | 2.3 (2.1-3.1) | 2.6 (2.0-3.2) | 0.832 |
| Albumin, g/L, median (IQR) | 40 (36-44) | 37 (32-41) | < 0.001 |
| Fluid indicators within 72 hours |  |  |  |
| Total fluid input (with enteral nutrition) within 72 hours, L, median (IQR) | 7.1 (6.1-8.1) | 7.3 (6.3-8.8) | 0.100 |
| Total fluid input (without enteral nutrition) within 72 hours, L, median (IQR) | 3.6 (2.6-4.6) | 3.8 (2.8-5.3) | 0.093 |
| Cumulative fluid balance within 72 hours, L, mean ± SD | 1.6 ± 1.3 | 1.9 ± 1.8 | 0.121 |
| Vasopressors, n (%) | 4 (1.7) | 27 (16) | < 0.001 |
| Mechanical ventilation, n (%) | 25 (10.6) | 67 (39.6) | < 0.001 |
| Acute Kidney Injury, n (%) | 12 (5.1) | 26 (15.4) | < 0.001 |
| Chloride indicators |  |  |  |
| New-onset hyperchloremia, n (%) | 12 (5.1) | 26 (15.4) | < 0.001 |
| [Cl^-^]_0_, mmol/L, median (IQR) | 103 (100-106) | 104 (100-107) | 0.221 |
| [Cl^-^]_max_, mmol/L, median (IQR) | 106 (102-111) | 104 (102-107) | 0.002 |
| Δ[Cl^-^] ≥ 5 mmol/L, n (%) | 53 (22.5) | 57 (33.7) | 0.008 |

HC, hyperchloremia ([Cl^-^] ≥ 110 mmol/L); SD, standard deviation; BE, base excess; GCS, Glasgow coma scale; APACHE II, acute physiology and chronic health evaluation II; SOFA, sequential organ failure assessment; IQR, interquartile range.
